# Did aid to the Ebola crisis divert aid for reproductive, maternal, and newborn health? An analysis of donor-reported data in Sierra Leone

**DOI:** 10.1186/s13031-024-00589-2

**Published:** 2024-04-27

**Authors:** Susannah H. Mayhew, Kirkley Doyle, Lawrence S. Babawo, Esther Mokuwa, Hana Rohan, Melisa Martinez-Alverez, Josephine Borghi, Ebola Gbalo Research Team, Dina Balabanova, Johanna Hanefeld, Tommy M Hanson, Bashiru Koroma, Gelejimah Alfred Mokuwa, Melissa Parker, Paul Richards, Ahmed Vandi, Catherine Pitt

**Affiliations:** 1 Department of Global Health and Development, London School of Hygiene & Tropical Medicine, London, UK; 2 Adjunct Professor, Njala University, Bo, Sierra Leone; 3 Independent Consultant, Washington DC, USA; 4 Department of Nursing, School of Community Health Sciences, Njala University, Bo, Sierra Leone; 5 Department of Public Health, Faculty of Health Sciences and Disaster Management, Eastern Technical University, Kenema, Sierra Leone; 6 Mattru School of Nursing, Bonthe District, Mattru, Sierra Leone; 7 University of Wageningen, Wageningen, The Netherlands; 8 Non-resident affiliate of the Center for Global Health Science and Security at Georgetown University, Washington DC, USA; 9 Independent Consultant, London, UK

**Keywords:** Development assistance for health, Official development assistance, Donor aid, Ebola, Reproductive health, Maternal and newborn health, Sierra Leone, West Africa, Aid tracking, Fungibility, Health financing, External financing

## Abstract

**Background:**

Infectious disease outbreaks like Ebola and Covid-19 are increasing in frequency. They may harm reproductive, maternal and newborn health (RMNH) directly and indirectly. Sierra Leone experienced a sharp deterioration of RMNH during the 2014–16 Ebola epidemic. One possible explanation is that donor funding may have been diverted away from RMNH to the Ebola response.

**Methods:**

We analysed donor-reported data from the Organisation for Economic Cooperation and Development (OECD)’s Creditor Reported System (CRS) data for Sierra Leone before, during and after the 2014–16 Ebola epidemic to understand whether aid flows for Ebola displaced aid for RMNH. We estimated aid for Ebola using key term searches and manual review of CRS records. We estimated aid for RMNH by applying the Muskoka-2 algorithm to the CRS and analysing CRS purpose codes.

**Results:**

We find substantial increases in aid to Sierra Leone (from $484 million in 2013 to $1 billion at the height of the epidemic in 2015), most of which was earmarked for the Ebola response. Overall, Ebola aid was additional to RMNH funding. RMNH aid was sustained during the epidemic (at $42 m per year) and peaked immediately after (at $77 m in 2016). There is some evidence of a small displacement of RMNH aid from the UK during the period when its Ebola funding increased.

**Conclusions:**

Modest changes to RMNH donor aid patterns are insufficient to explain the severe decline in RMNH indicators recorded during the outbreak. Our findings therefore suggest the need for substantial increases in routine aid to ensure that basic RMNH services and infrastructure are strong *before* an epidemic occurs, as well as increased aid for RMNH *during* epidemics like Ebola and Covid-19, if reproductive, maternal and newborn healthcare is to be maintained at pre-epidemic levels.

**Supplementary Information:**

The online version contains supplementary material available at 10.1186/s13031-024-00589-2.

## Introduction

Infectious disease epidemics – like Ebola and Covid-19 – are increasing in frequency [1]. These epidemics harm reproductive, maternal, and newborn health (RMNH) both directly (affecting women and children’s health) and indirectly (by disrupting critical services and potentially diverting international aid and domestic expenditure for RMNH services) [2–5]. While research is beginning to quantify the negative impacts of epidemics on RMNH, little is known about the effect of such outbreaks on funding flows, in particular, whether crisis-funding for outbreaks may displace regular aid for RMNH.

This question is important because international aid continues to provide a large proportion of health expenditure in the world’s poorest countries. In 2020, aid accounted for 29% of health expenditure in low-income countries [6]. The need for sustained aid contributions to improve RMNH in low-income countries is well established [7, 8], yet aid for RMNH has been falling globally since 2017, particularly for reproductive health services for non-pregnant women [9]. Moreover, total aid is predicted to decline further over the next five years as countries prioritise their own domestic responses to the Covid-19 pandemic [10]. Funding flows for epidemic response can be hard to establish, but are often very large [11, 12]. Previous work has provided some estimates of Ebola aid volumes [11] and other work has looked at whether donor aid in general displaces domestic spending but does not consider donor aid for Ebola [9, 12]. No research has examined the impact, or displacement effects, of funding flows for disease outbreaks on RMNH funding.

We address this gap by examining international funding flows before, during and after the Ebola outbreak in Sierra Leone. Despite pre-Ebola gains in RMNH outcomes, Sierra Leone’s maternal and neonatal mortality severely worsened during the epidemic. These mortality rates only started to recover from 2020 (five years after the epidemic) after a concerted joint effort by Unicef and others, and remain far short of SDG targets [10, 13, 14]. Audit reports from Sierra Leone show that health expenditure in 2014 (the first full year of the Ebola outbreak) was almost double that of 2013 (and preceding years), and donors provided around 80% of 2014 expenditure [15]. We quantified aid flows to Sierra Leone for the Ebola response and examined whether there was any evidence that these flows showed a reallocation of funding away from RMNH services in order to fund the Ebola response (i.e. displacement). Understanding what happened to international aid for RMNH in Sierra Leone is an important first step to understanding how RMNH activities can be protected during future epidemics.

### Sierra Leone, Ebola, and Reproductive, Maternal and Newborn Health

Despite a turbulent post-independence history in which the country suffered decades of brutal civil war and aid instability [16], Sierra Leone made significant progress on improving RMNH between 2002 and 2014 [8, 17, 18].

In May 2014, the first case of Ebola was formally identified in Sierra Leone [19]. Two months later, the president declared a state of emergency and Sierra Leone battled Ebola until March 2016 when it was declared Ebola-free [19, 20]. In just under two years, Ebola infected over 14,000 people across Sierra Leone, killing 3,956 [20]. The already fragile health system was overwhelmed by the task of treating and containing Ebola. Healthcare visits for non-Ebola conditions plummeted and health care workers were unable or unwilling to provide routine health services, prompting an increase in deaths due to other causes [21, 22]. Pregnant women, infants, and children comprised a significant portion of these indirect, crisis-related deaths [5, 23, 24], with maternal mortality becoming highest in the world, with an estimated 1,120 deaths per 100,000 live births in 2017 [25] compared to 857/100,000 live births in 2008 [26].

The precise pathways by which the Ebola crisis contributed to these deaths remains unclear, which hampers efforts to protect RMNH from harm in future outbreaks. One hypothesis, which we examine, is that aid earmarked for Ebola may have been reallocated away from other priorities like RMNH, thus resulting in a “displacement effect”. 

## Methods

### Patient and public involvement

No patients were involved in this study which utilises only publicly available secondary data on aid flows, reported in the Organization for Economic Cooperation and Development’s (OECD) Creditor Reporting System (CRS) database.

The questions this study seeks to answer were developed with partners at Njala University in Sierra Leone and are informed by previous work on Ebola responses in early affected districts.

### Data sources

We analysed aid flows in the Organization for Economic Cooperation and Development’s (OECD) Creditor Reporting System (CRS) aid activities database using the February 2021 data update, which includes the relevant years for our analysis. The CRS contains data reported by 138 donors: 50 bilateral (i.e. country) donors, 49 multilateral institutions, and 39 private donors, although more of these donors have reported their aid for recent years (127 for 2019) than prior years (54 reported for 2010).

The CRS contains 87 variables, including: calendar year, donor, disbursement amount, flow type, recipient, sector, purpose, project title, short and long description of the funded activities. The CRS avoids double-counting the aid from bilateral donors (e.g. US or UK) that flows to and through multilateral donors (e.g. European Union or the United Nations Population Fund) by identifying the “donor” of a particular aid flow as either a bilateral or multilateral institution (not both), depending on which retained control over the recipient and purpose of the funds. We analyse the disbursement value – the “actual international transfer of financial resources, or of goods or services” [27] – rather than commitments, which may not reflect actual transfers. Aid flows include overseas development assistance (ODA) loans and grants, as well as grants from private donors; we excluded equity investments and other official flows, consistent with past analyses [28]. Recipients are countries, regions, or unspecified; more than a quarter of global aid for RMNH is categorised as flowing to regional and unspecified recipients [28]. Each aid record is categorised into a single sector, and within each sector, into a specific purpose. Health sector purposes include (among others) infectious disease control, reproductive healthcare, and family planning. While most aid for health activities is reported within the health sector, the humanitarian sector also includes aid for health alongside other types of humanitarian activities. In addition, free text fields – of varying length and quality – describe the activities.

### Categorization of aid for RMNH and for Ebola

#### Identification of aid for reproductive, maternal, and newborn health

We used Muskoka2 estimates of aid for RMNH, which are generated by applying an algorithm to the CRS database, as described in detail elsewhere [28]. Muskoka2 aid for RMNH estimates reflect aid for the reproductive health of non-pregnant women, maternal health, and the health of babies aged up to 28 days. The estimates include most of the aid categorised in the CRS’s reproductive health and family planning purpose codes, as well as relevant shares of aid for health systems, basic health services, malaria, HIV, water and sanitation, and the humanitarian sectors. We chose not to include child health because of difficulties in disentangling aid for child health from aid for Ebola. In the aid activities database, there are separate purpose codes for reproductive health and family planning, but no purpose codes specific to child health. A proportion of child health support can be assumed through general purpose codes like health systems, basic health services, malaria and other infectious diseases – which include Ebola – but these are very difficult to disentangle. We therefore focus solely on reproductive, maternal and new-born health (RMNH).

In addition, as a sensitivity analysis and to explore our data in greater depth, we also employed an alternative, narrower approach, by which we only examined aid categorised in the “reproductive health” and “family planning” (RH + FP) purpose codes within the CRS.

#### Identification of aid for Ebola

To identify aid for Ebola, we first implemented a key term search for the terms “Ebola”, “EVD”, “Zoonotic”, “Haemorrhag”, and “Hemorrhag” (not case sensitive). We then manually classified the identified records, retaining only those that were directed towards understanding, treating, preventing transmission, and supporting those with or directly affected by Ebola (e.g. including social services for children whose parents died of Ebola). We included aid for unspecified zoonotic diseases or haemorrhagic fevers which could include Ebola. We excluded aid which mentioned “Ebola” in describing the context of the activities (e.g. “post Ebola recovery phase”) if the activities themselves did not focus on understanding, treating, or preventing Ebola; for example, activities for rebuilding basic health and other services in the post-crisis phase were excluded as not being “aid for Ebola.” A list of excluded records is provided in Annex 1.

### Data analysis

We analysed aid to Sierra Leone. We also examined aid to sub-Saharan Africa and West Africa (combined), as well as aid for unspecified recipients, because a portion of aid for these recipients may ultimately flow to Sierra Leone. We did not make any assumptions about what shares of this regional and unspecified aid may have benefitted Sierra Leone and instead present this aid separately (Annex 2).

We analysed trends in total aid, as aid for both Ebola and RMNH cut across health, humanitarian, and other sectors. We examined the period 2010–2019: four years before the Ebola crisis began and 3 full years after it ended. To avoid reporting bias (because increasing numbers of donors report their aid each year), analyses were restricted to aid from the 53 donors reporting any disbursements to the CRS in both 2010 and 2019.

To understand whether donors may have reallocated aid away from RMNH and towards Ebola, we began by describing aggregate levels and trends in aid for Ebola, aid for RMNH, and total aid. We identified the top 5 donors providing aid for RMNH and (separately) for Ebola in Sierra Leone over the period (2010–19). The top 5 donors for RMNH over the period of analysis were (in order of magnitude) the UK, Global Fund, US, EU, and Germany, and of these, only 3 (UK, US and EU) provided any aid for Ebola and were therefore eligible for analysis on displacement effect. For each of these three donors we examined their overall aid levels, and the composition of their aid over 2002–19. This longer-term perspective allowed us to explore whether year-on-year fluctuations were out of step with each donor’s previous pattern of disbursements; this analysis was possible because – unlike many other donors – they reported aid from 2002 onwards. Amounts are presented in constant 2018 US dollars based on the OECD’s development assistance committee deflators, which adjust for inflation and variations in exchange rates.

## Results

### Trends in total aid, aid for Ebola, and aid for RMNH

**Fig. 1:**
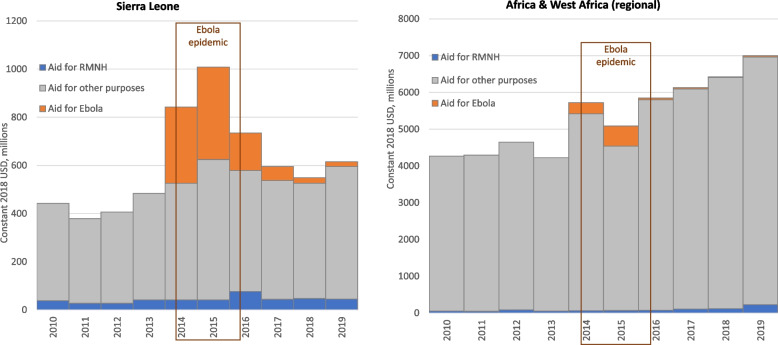
Total aid to Sierra Leone, 2010–19: Ebola, RMNH, and other purposes. Source/Notes: Authors’ analysis of the OECD’s CRS dataset

Annual total aid to Sierra Leone (Fig. 1) was more than twice as high in 2014 ($843 m) and 2015 ($1,008 m) – the height of the Ebola crisis – compared to 2010–13 (mean: $428 m per year, sd: $39 m). Total aid decreased in 2016 ($735 m) and again in 2017 ($595 m), but remained at higher than pre-Ebola levels.

Aid for Ebola to Sierra Leone amounted to $958 m over 2014–19. It comprised 38% of Sierra Leone’s total aid both in 2014 ($317 m) and in 2015 ($384 m), having been zero in prior years. Ebola aid for Sierra Leone dropped in 2016 (to $156 m, 21% of total), and continued to decline sharply in subsequent years, with just $20 m (3% of total) disbursed in 2019, the most recent year of analysis. Regional (i.e. multi-country) Ebola aid for West Africa and Africa combined – which may support activities in Sierra Leone and elsewhere – increased from $0.4 m in 2013 to $304 m in 2014 and $558 m in 2015, before falling to $47 m in 2016 and continuing at lower levels through 2019.

RMNH aid constituted a small proportion of total aid to Sierra Leone (range: 4%-10%). It remained constant over the period 2013–2015 (at $42 m; similar to the 2010 level, $39 m) when estimated using the Muskoka2 method, and surged by 83% to $77 m in 2016 – the year the Ebola epidemic ended – before returning in 2017–19 to pre-epidemic levels (range: $44 m—$47 m). RMNH aid constituted 1%—2% of total aid for the West Africa and Africa regions (range: $50 m to $76 m) until 2017 (no marked change in the 2014–15 period), when it began to increase, reaching $227 m (3%) in 2019. A further detailed breakdown of data is provided in the supplementary Figures in Annex 2.

### Top 5 donors of aid for Ebola and for RMNH to Sierra Leone

**Fig. 2:**
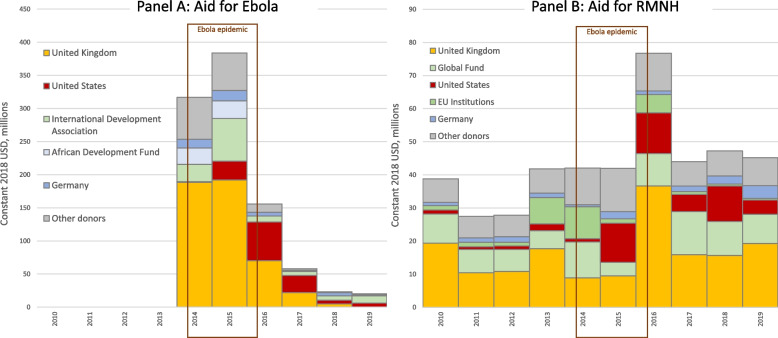
Aid for Ebola and RMNH to Sierra Leone by donor (top 5), 2010–2019. Source/Notes: Authors’ analysis of the OECD’s CRS dataset. Lefthand panel presents aid for Ebola. Righthand panel presents aid for reproductive, maternal, and newborn health (RMNH). Panels are presented on different y-axes

Between 2010–2019 Sierra Leone’s leading Ebola aid donor was the UK ($478 m), which provided 50% of the country’s total Ebola aid, followed by the United States ($125 m, 13%) and the International Development Association (IDA: World Bank Group, $124 m, 13%) (Fig. 2). In 2014, the first year of the outbreak, the UK provided 60% of Ebola aid to Sierra Leone ($189 m), sustaining the amount in 2015 before gradually reducing. The USA only played a substantive role from 2015 onwards and the IDA gave substantial funds only in 2014–15. The African Development Fund ($43 m, 4%) and Germany ($43 m, 4%) were the fourth and fifth largest donors of Ebola aid to Sierra Leone.

For RMNH, the UK was again the largest donor to Sierra Leone in the pre-epidemic period (2010–13, $58 m, 43%) and overall (2010–19, $164 m, 38%). The Global Fund was Sierra Leone’s second-largest RMNH donor across the 2010–19 period, disbursing $84 m (20% of Sierra Leone’s RMNH aid), of which 75% reflected aid directed towards sexually transmitted infections including HIV/AIDS. The United States was the third largest RMNH donor, disbursing $50 m (12%) of RMNH aid across the decade, despite having provided minimal aid for RMNH in Sierra Leone in the pre-epidemic period (2010–13, $5 m, 4%). The EU ranked fourth providing $30 m (7% all RMNH aid 2010–19). Having provided very little aid for RMNH in 2010–12, the EU provided substantial funding in 2013 ($8 m, 19% of aid for RMNH that year), which it further increased in the first year of the epidemic, 2014 ($10 m, 23%), but then provided virtually no funding for RMNH in 2015 ($1 m, 3%), returning in 2016 with less, though still quite substantial funding ($6 m, 7%). The fifth RMNH donor, Germany, disbursed $17 m (4%) in RMNH aid to Sierra Leone over the 2010–19 period with a mean of $1.3 m per year between 2010–2016 with small variations (dip in 2014, rise in 2015), rising to a high of $3.9 m only after the epidemic in 2019. The overall spike (just for one year) observed in aid for RMNH in 2016 was driven by a combination of substantial and sustained aid from the US from 2015 into 2016, a return of aid from the EU to a similar magnitude of that in 2014, and a very large increase in aid from the UK. A further detailed breakdown of data is provided in the supplementary Figures in Annex 2.

### Aid to Sierra Leone from the UK, USA and EU

**Fig. 3:**
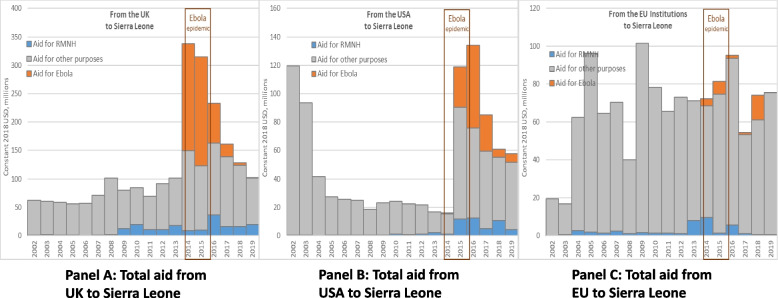
Total aid to Sierra Leone from the UK, USA and EU, 2002–2019: Aid for Ebola, aid for RMNH, and aid for other purposes. Source/Notes: Authors’ analysis of the OECD’s CRS dataset. Panels are presented on different y-axes

To examine whether the Ebola aid provided by the UK, USA and EU was additional to or displaced their aid for RMNH (Fig. 3), we explored these donors’ aid in greater depth. These were the only donors among the top 5 for both RMNH and Ebola over the decade.

In 2014, the first year of Sierra Leone’s Ebola epidemic, the UK’s aid for RMNH fell to $9 m, a 50% ($9 m) reduction relative to 2013; however, the 2014 value was only 18% ($2 m) lower than in 2012 and was substantially higher than the UK’s aid for RMNH in 2002–8 (range: $0 m—$2 m). In 2015, the second year of the epidemic, the UK only increased its aid for RMNH by 7% relative to 2014 (to $10 m), meaning it remained 46% ($9 m) below 2013 levels. In 2016, however, the year the epidemic ended, the UK increased its aid for RMNH nearly four-fold, to $37 m. There is therefore some evidence that the UK’s $189 m contribution to Ebola in 2014 and $192 m in 2015 may have displaced up to half of its aid for RMNH in the first two calendar years of the epidemic. The accumulated $18 m shortfall seems, however, to have been “replenished” in 2016.

The USA, by contrast, provided very little aid (≤ $2 m annually) to Sierra Leone for RMNH in 2002–13, meaning that there was minimal scope for displacement. In 2014, the first year of the epidemic, the USA’s aid for RMNH (as well as aid for Ebola and overall aid) remained at comparably low levels to previous years. From 2014 to 2015, the second year of the epidemic, the USA simultaneously increased its aid for RMNH from $1 m to $12 m, increased its aid for Ebola from $1 m to $29 m, and also increased its aid for other purposes from $14 m to $79 m. In 2016, the USA’s aid for RMNH remained at $12 m, aid for Ebola doubled to $58 m, and aid for other purposes fell by 20%. In 2015 and 2016, the USA’s total aid for Sierra Leone ($119 m and $134 m) was higher than it had been in any year since 2002 ($120 m), following the civil war.

Aid for RMNH from the EU averaged $1.4 m annually in the years 2002–12, before a sudden increase to $8 m in 2013 – the year before the outbreak – and $10 m in 2014. The drop to $1.4 m in aid for RMNH in 2015 was accompanied by increases in aid for Ebola (from $4 m in 2014 to $7 m in 2015) and in aid for other purposes (from $59 m in 2014 to $73 m in 2015). In 2016, aid for RMNH increased again, to $6 m but was < $1 m annually in 2017–19. Viewed over the 18-year period analysed, the high levels of aid for RMNH in 2013, 2014, and 2016 appear more anomalous than the drop in 2015.

## Discussion

Our analysis shows that Ebola aid was to a large extent additional to aid for RMNH, which was sustained overall through the Ebola outbreak; however, the UK was Sierra Leone’s largest donor and we found evidence of a short-term displacement of its aid for RMNH during the outbreak, which was mitigated by increases from other donors. There is apparently a short-term displacement effect in EU aid for RMNH too, but their RMNH funding fluctuates too much to draw any firm conclusions. Total aid to Sierra Leone increased very substantially during the Ebola epidemic years 2014–15 and into 2016, peaking at over $1 billion in 2015. For just one year – 2016 – at the end of the epidemic, RMNH aid almost doubled. This surge was driven by the more than tripling of UK RMNH aid in 2016 and substantial contributions from the USA and the EU. One explanation for the spike in 2016 may be that donors became aware of the spiralling maternal and neonatal mortality and morbidity rates and sought belatedly to intervene. Another explanation may be related to the effective collapse of the Sierra Leone government supply chain agency; the UK’s Department for International Development (DFID, now FCDO) stepped in to procure RMNH products that year, and subsequently channelled its funding to systems support, which indirectly benefitted RMNH.

To our knowledge, this analysis is the first to assess the impact of aid for an infectious disease epidemic on aid for RMNH. Our estimates of Ebola aid for Sierra Leone are higher than reported in a study of funding flows for Ebola and Zika [11], and broadly similar overall to estimates produced by the Institute for Health Metrics and Evaluation [29]. Whereas the literature on fungibility explores whether donor aid flows displace domestic expenditure [30], we have focused on donor behaviour and whether donors’ emergency response to disease outbreaks comes at the expense of their support for RMNH. An analysis of aid for Syria during the recent conflict found that humanitarian aid may have had a small displacement effect on development aid for the health sector, although this may reflect a relabelling of similar activities and both humanitarian and health sector aid increased over the study period [31].

In Sierra Leone, the changes in sources – but not overall levels – of aid for RMNH during the epidemic cannot explain the severe decline in RMNH indicators. Other causal pathways must therefore be considered. Ebola decreased utilisation of health services [13, 14, 32, 33]; one estimate indicated that excess maternal and newborn mortality through non-utilisation of services during the outbreak (at least 3,600 deaths) was almost as large as the numbers of people who died from Ebola (3,956) [5]. For women and neonates who did use services, institutional death rates increased [13, 14] both as a direct effect of Ebola (higher foetal loss and pregnancy-associated haemorrhage) [34] and because of poor supplies and equipment or inadequate workforce [22, 35, 36].

Future research should explore patterns of domestic spending on RMNH before, during and after the Ebola crisis. Co-author LB, who was part of Sierra Leone’s Ebola response, notes that in the early stages of the outbreak, almost all programme activities of the Ministry of Health and Sanitation were halted in favour of outbreak activities, consequently, RMNH activities noticeably decreased. There has long been debate about whether and to what extent donor funding should supplement domestic spending on health (so-called “additionality”) [9]. Robust analysis of domestic spending, comparing to our donor reported spending, would inform discussions on whether donors should support government priorities during outbreaks (e.g. to fund outbreak response) or concentrate their efforts instead on the services left behind (like RMNH).

### Limitations

We acknowledge several limitations of the data and its analysis. The quality of donors’ reported data varies between donors and over time, and donors’ categorisation of their aid by sector and purpose may not be consistent. The CRS only includes data from those donors who report to the system, which does not currently include China and Brazil, and the time periods for which data are available are similarly restricted. Limitations of our analysis include the use of key terms and manual coding, which may lead to some degree of misclassification. It is noted that funds disbursed by donors do not necessarily reach the ground. In Sierra Leone, corruption is a particular concern and the Government’s audit reports identified substantial missing Ebola aid [37]. Our analysis does not include domestic spending, so does not represent the total health expenditure (however during the Ebola epidemic donors provided around 80% total health expenditure [15, 37]).

## Conclusion

Our findings have implications for protecting RMNH during future epidemics and pandemics. First, sustaining pre-crisis services and outcomes during an outbreak requires *increased* RMNH funding *during* the outbreak to support continuity of services, staff and equipment. However, significant influxes of humanitarian aid, even when additional to regular RMNH aid, are insufficient to protect RMNH outcomes in times of crisis. The lack of a strong, resilient health system in Sierra Leone is highlighted as one reason why the impact of the Ebola outbreak on routine services was so catastrophic [36, 38]. Second, greater investment is therefore required in routine services and infrastructure as part of pandemic preparedness, to build health systems with the strength and resilience to withstand shocks and protect RMNH [12, 39, 40]. Covid-19 led to a marked increase in development assistance for health and this represents an unprecedented opportunity to sustain funding for pandemic preparedness which must include RMNH [12]; some authors also call for using humanitarian aid in this endeavour [41].

### Supplementary Information

**Additional file 1:** **Annex 1.** List of records excluded from Ebola categorization. **Annex 2.** Detailed breakdown of all sector data: aid to Sierra Leone, Africa and West Africa region. **Fig. S1.** Total aid for all sectors by sector. **Fig. S2.** Total aid to Sierra Leone, Africa and West Africa, 2010-19: Ebola, RMNH, and other purposes. **Fig. S3.** Aid for Ebola by sector. **Fig. S4. **Aid for Ebola and RMNH to Sierra Leone, & Africa/West Africa, by donor (top 5), 2010-2019. **Fig. S5.** Aid for RMNH to Sierra Leone and Africa/West Africa, 2010-2019, disaggregated by components.

## Data Availability

All data generated or analysed during this study are included in this published article and its supplementary information files.
