# Supplementary material for: Did aid to the Ebola crisis divert aid for reproductive, maternal, and newborn health? An analysis of donor-reported data in Sierra Leone
Source: Confl Health. 2024 Apr 27;18:38. doi: 10.1186/s13031-024-00589-2 (PMC11055248; doi:10.1186/s13031-024-00589-2)

**Did aid to the Ebola crisis divert aid for reproductive, maternal, and newborn health? An analysis of donor-reported data in Sierra Leone.**

**Supplementary Information**

**ANNEX ONE: EXCLUDED RECORDS**

- “SIERRA LEONE DELIVERY UNIT PHASE II Sierra Leone Delivery Unit Phase II support the Government of Sierra Leone's efforts to recover from the Ebola crisis and accelerate development by adopting a new, results-focused approach to delivering the most important priorities.”
- “SIERRA LEONE EARLY RECOVERY ON HEALTH AND WASH Sierra Leone Early Recovery on Health and WASH To support early recovery from the effects of Ebola through supporting basic services and addressing urgent needs in Sierra Leone in 2015”
- “STRENGTHENING THE HEALTH SYSTEM IN SIERRA LEONE POST EBOLA THROUGH SCALING UP A NEW MIDWIFERY SCHOOL IN BO DISTRICT / SOUTHERN PROVINCE Strengthening the health system in Sierra Leone Post Ebola through scaling up a new midwifery school in Bo district / Southern Province Strengthening the health system in Sierra Leone Post Ebola through scaling up a new midwifery school in Bo district / Southern Province”
- “NUFU AGREEMENT NUFU Agreement ZOOTOX-2007-11: Collaborative research in environmental toxicology and zoonotic diseases in the Human Domestic animal Wildlife interface areas of Eastern and Southern Africa - A South-North Veterinary Network”

**ANNEX 2: SUPPLEMENTARY FIGURES SHOWING DETAILED BREAKDOWN OF AID**

**Supplementary Figure S1.**

**Total aid for all sectors by sector**

The total value of aid to Sierra Leone nearly doubled from annual averages in the 2010-13 period to the 2 main years of the Ebola outbreak (2014-15). This increase was primarily driven by a dramatic increase in humanitarian aid, but also a very substantial increase in general budget support, and a lesser increase in aid categorised as being within the health sector. A substantial increase in humanitarian funding directed towards relevant African regions also occurred in 2014, but returned to pre-crisis levels the following year although total regional aid continued to increase to the end of the study period (2019).


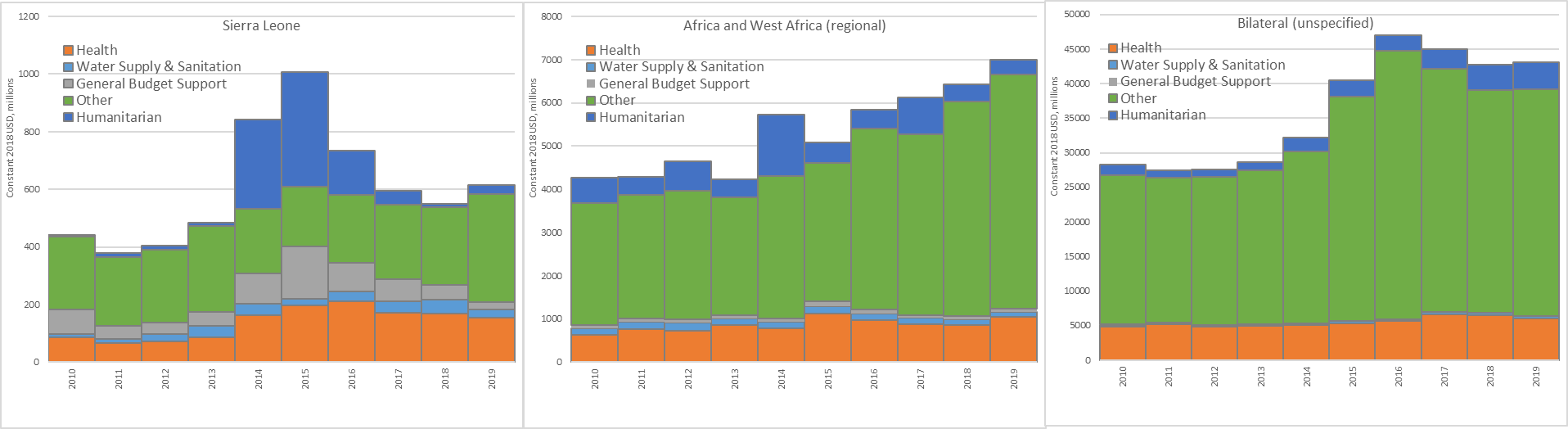


**Supplementary Figure S2.**

**Total aid to Sierra Leone, Africa and West Africa, 2010-19: Ebola, RMNH, and other purposes**

Upper panels (A) show aid for reproductive, maternal, and newborn health (RMNH) using the Muskoka2 method, a wider metric of aid for RMNH, in bright blue. Lower panels (B) show aid categorised in the CRS' reproductive health and family planning purpose codes in dark blue. RH+FP: Reproductive health and family planning purpose codes in the CRS dataset.


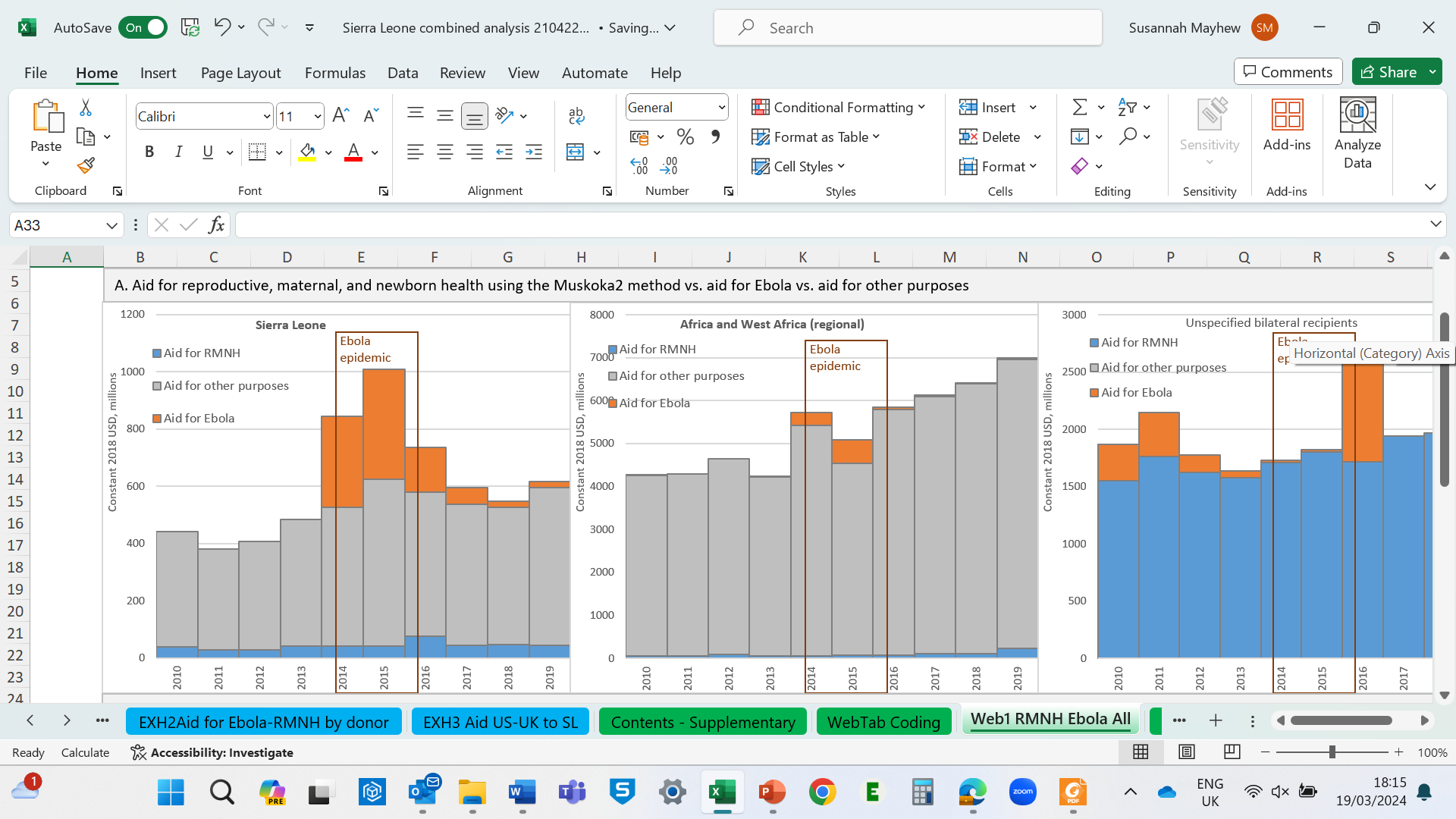

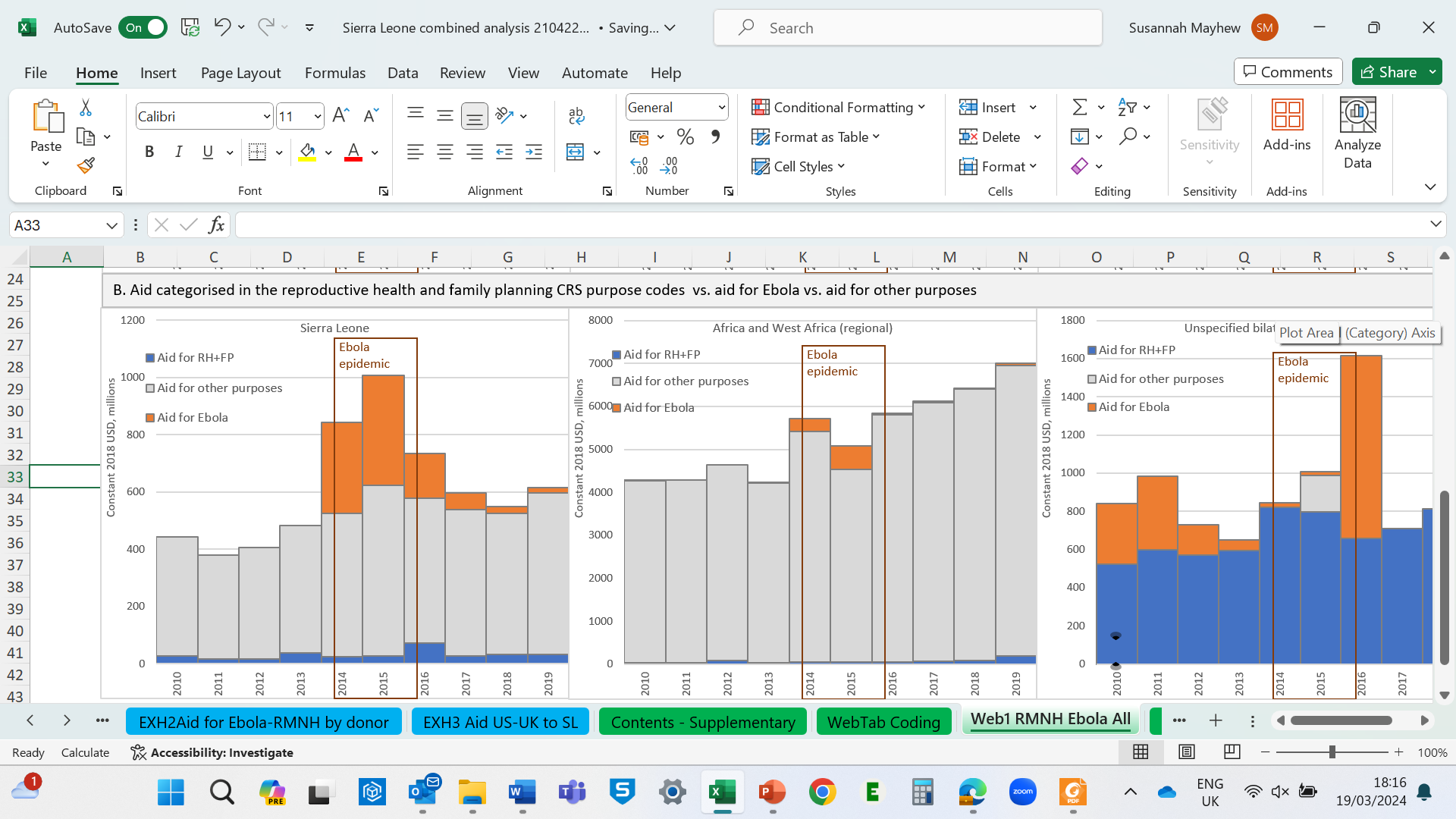


**Supplementary Figure S3.**

**Aid for Ebola by sector**

No aid was provided to Sierra Leone or relevant African regions which specifically mentioned Ebola or related terms in the period 2010-13. In that period, aid was provided to “South of Sahara, regional” by the EU Institutions, channelled through WHO, for $0.90m in 2008, $1.10m in 2010, and $0.36m in 2013, which was described as: “SUPPORT COUNTRIES EMERGENCY PREPAREDNESS AND RESPONSE TO MAJOR EPIDEMIC PRONE DISEASES IN WEST AND CENTRAL AFRICA Support countries emergency preparedness and response to major epidemic prone diseases in West and Central Africa The project enhances country capacity to detect and control epidemics of meningitis, yellow fever, Ebola, Marburg and plague in West Africa and Central Africa”. Other aid for Ebola before 2014 related to previous Ebola epidemics elsewhere in Africa and to efforts to improve medical training to address outbreaks.

In 2014, more than $300m was provided and in 2015, this aid for Ebola in Sierra Leone rose to over $400m. Most aid for Ebola was classified as falling within the humanitarian sector, although a substantial proportion – and most of the “bilateral, unspecified” funding – was classified within the health sector, largely in the purpose code for other infectious diseases (i.e. not malaria, HIV, or TB). Increased bilateral (unspecified) funding is seen through to the end of the study period (2019).


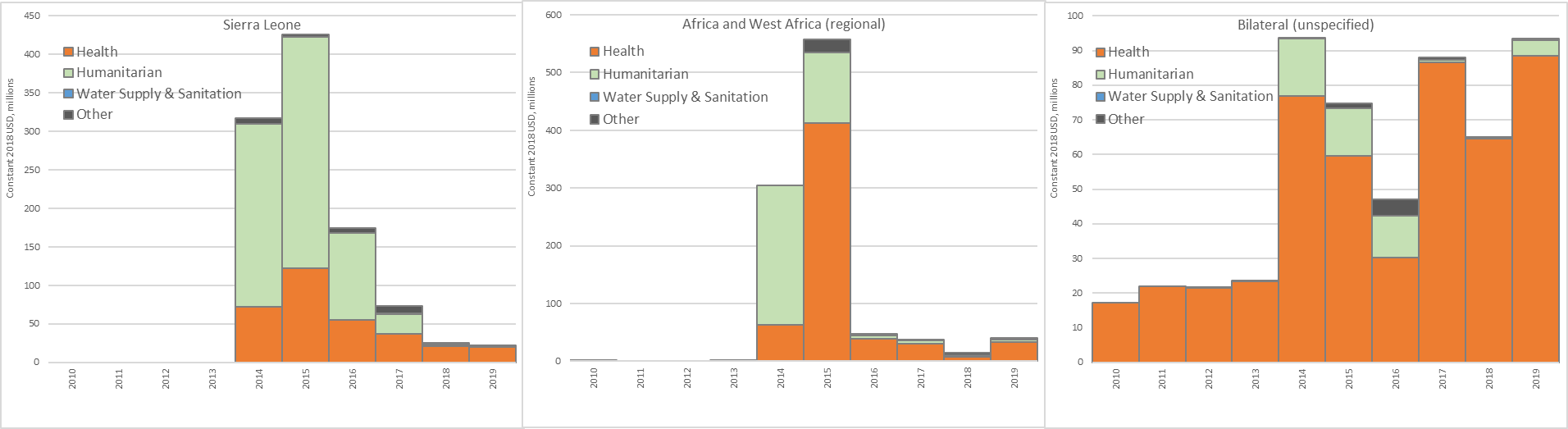


**Supplementary Figure S4.**

**Aid for Ebola and RMNH to Sierra Leone, & Africa/West Africa, by donor (top 5), 2010-2019**

Upper panels (A) show aid for Ebola. Central panels (B) show aid for reproductive, maternal, and newborn health (RMNH) using the Muskoka2 method, a wider metric of aid for RMNH, in bright blue. Lower panels (C) show aid categorised in the CRS' reproductive health and family planning purpose codes. RH+FP: Reproductive health and family planning purpose codes in the CRS dataset.


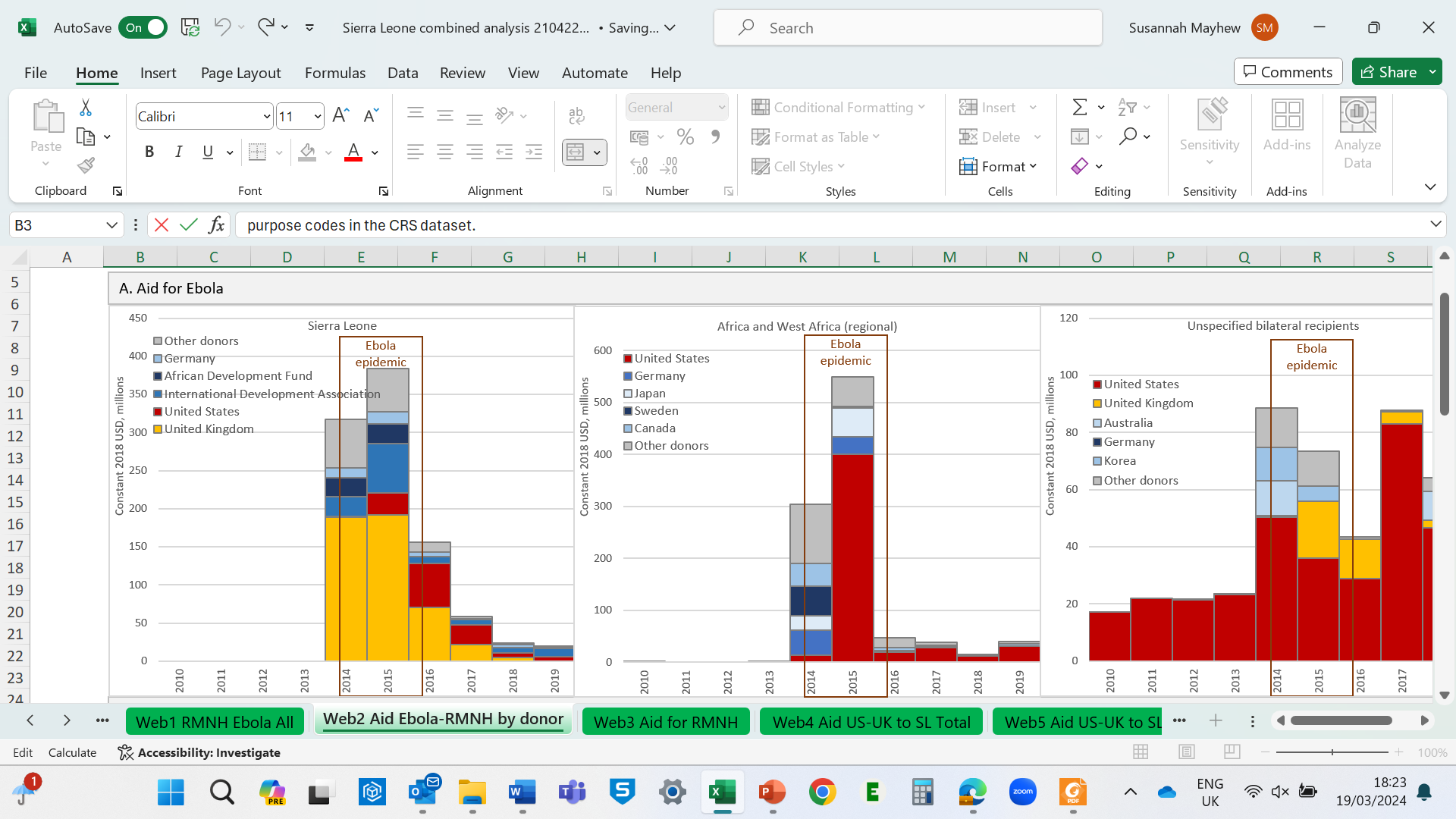


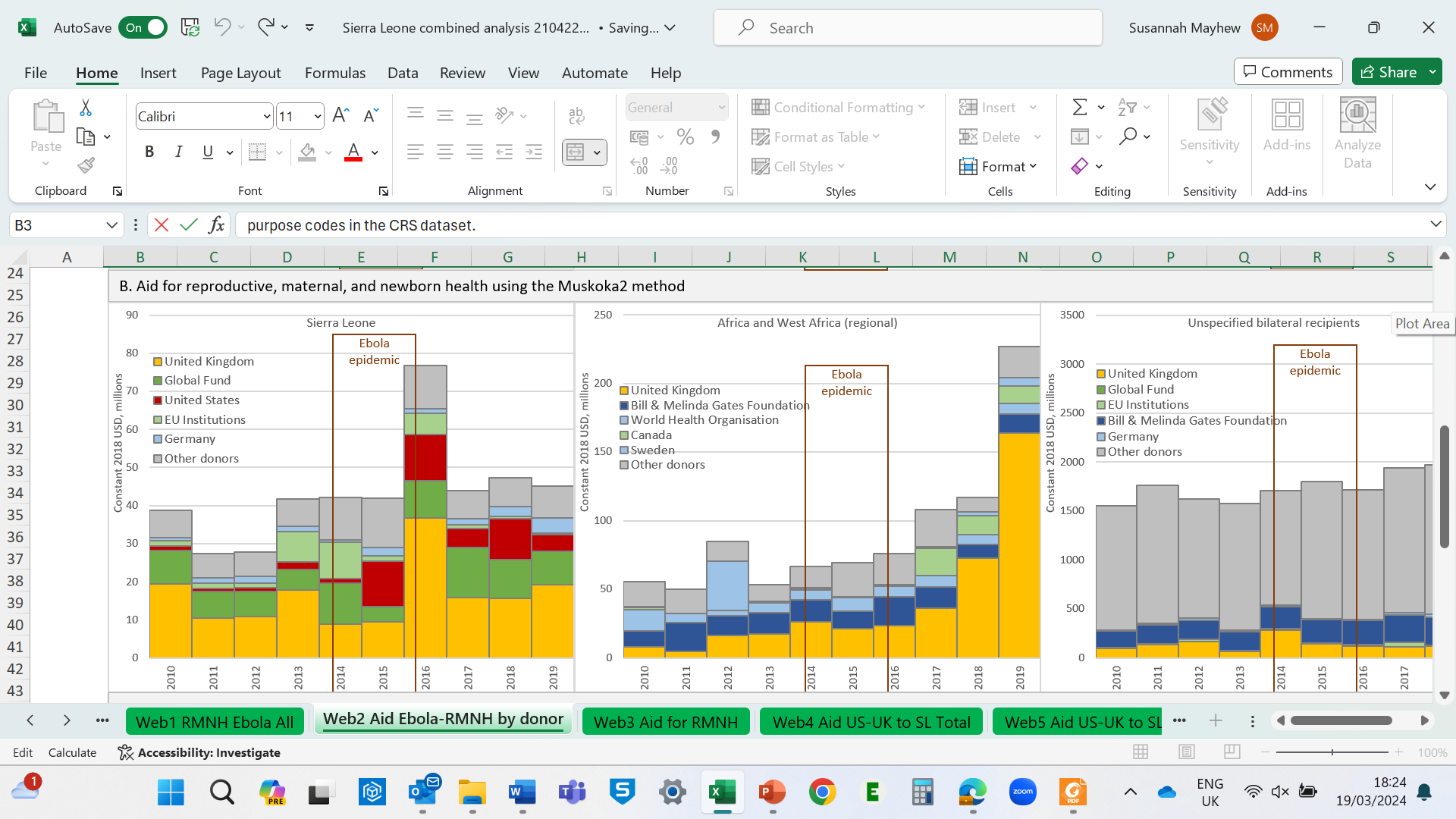


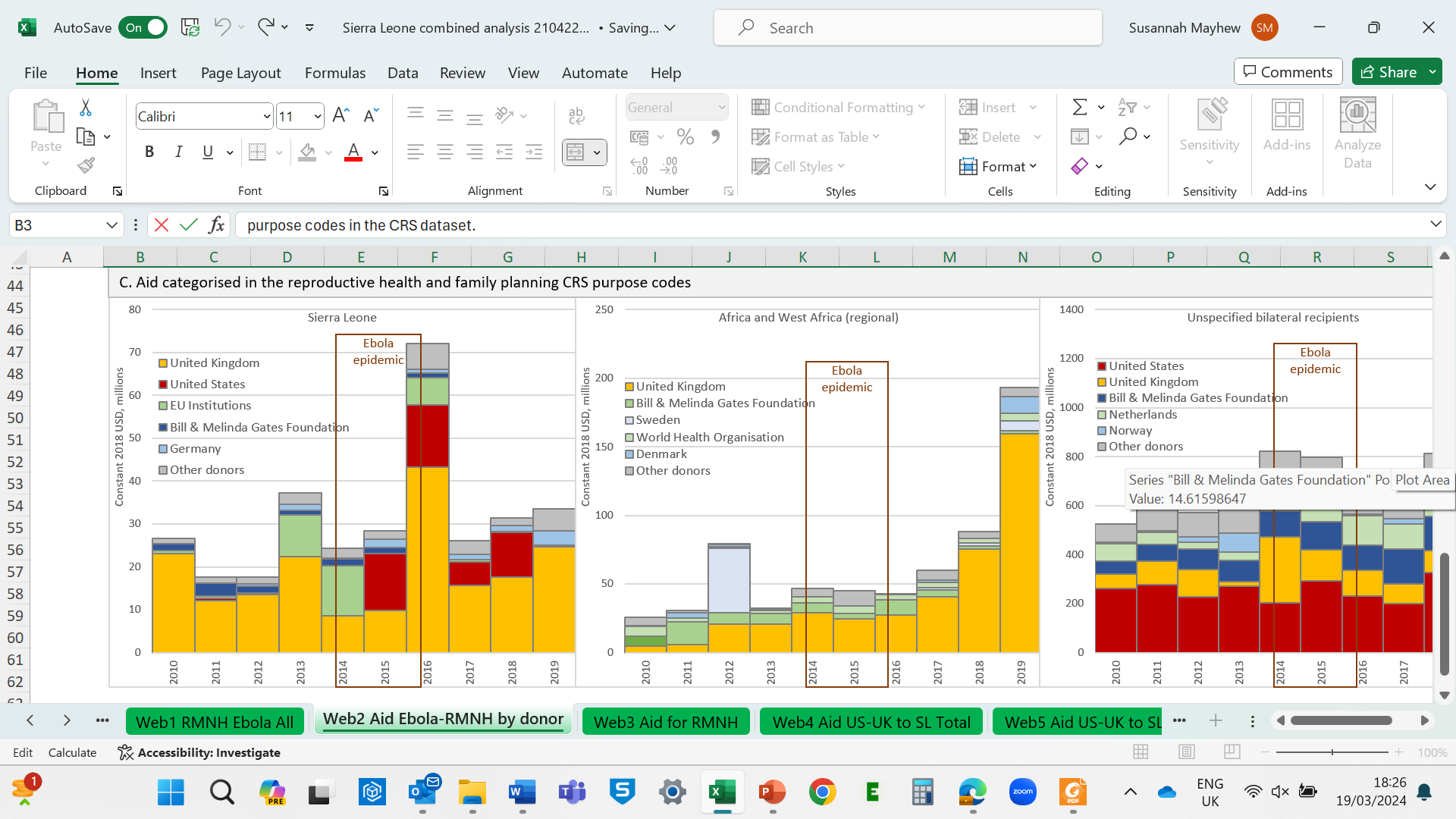


**Supplementary Figure S5.**

**Aid for RMNH to Sierra Leone and Africa/West Africa, 2010-2019, disaggregated by components**

Upper panels (A) show aid for reproductive, maternal, and newborn health (RMNH) using the Muskoka2 method, a wider metric of aid for RMNH, with totals disaggregated into aid for maternal and newborn health (grey) and aid for reproductive health of non-pregnant women (yellow). Lower panels (B) show aid categorised in the CRS' reproductive health (light blue) and family planning (orange) purpose codes. RH+FP: Reproductive health and family planning purpose codes in the CRS dataset.


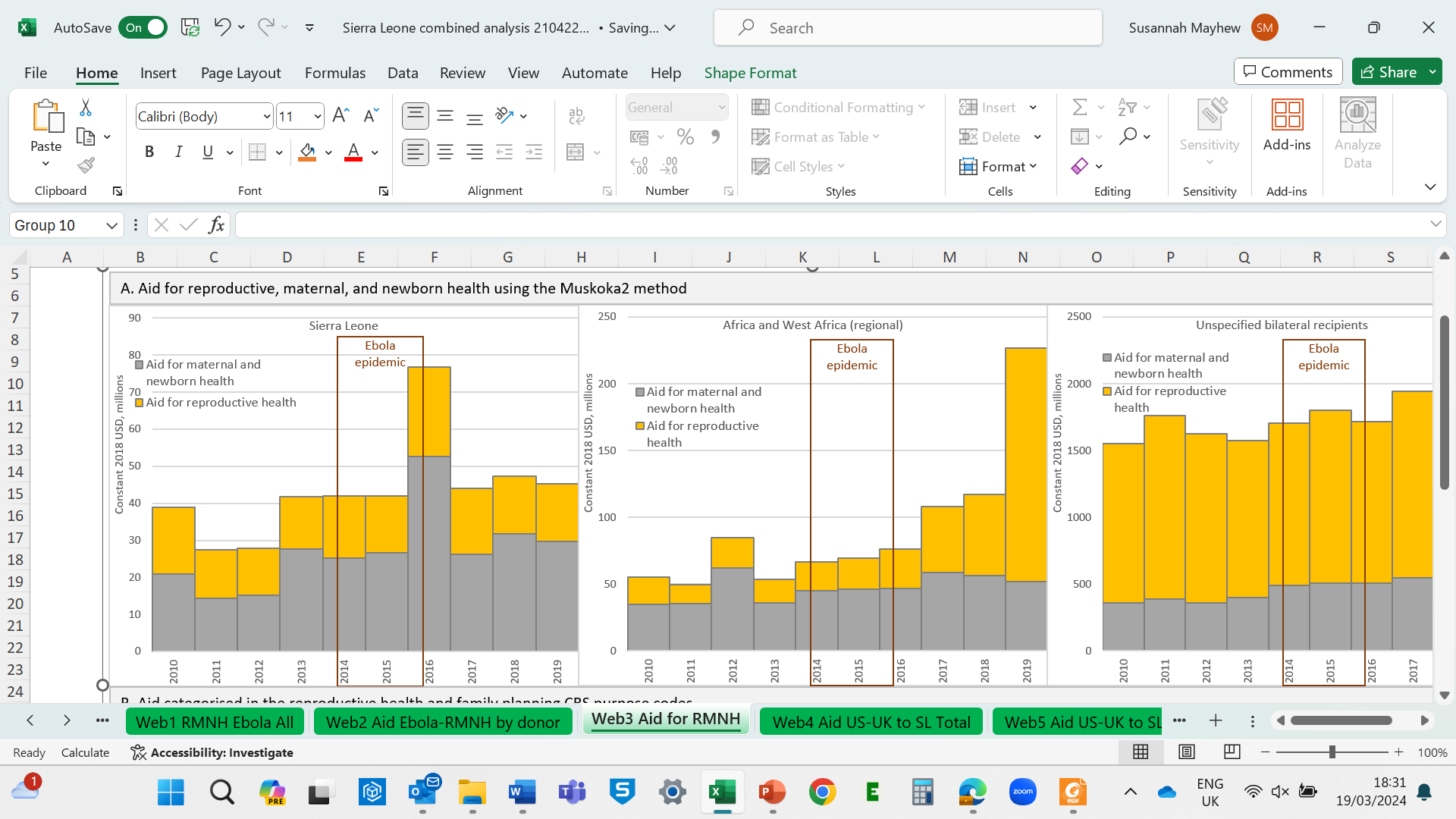


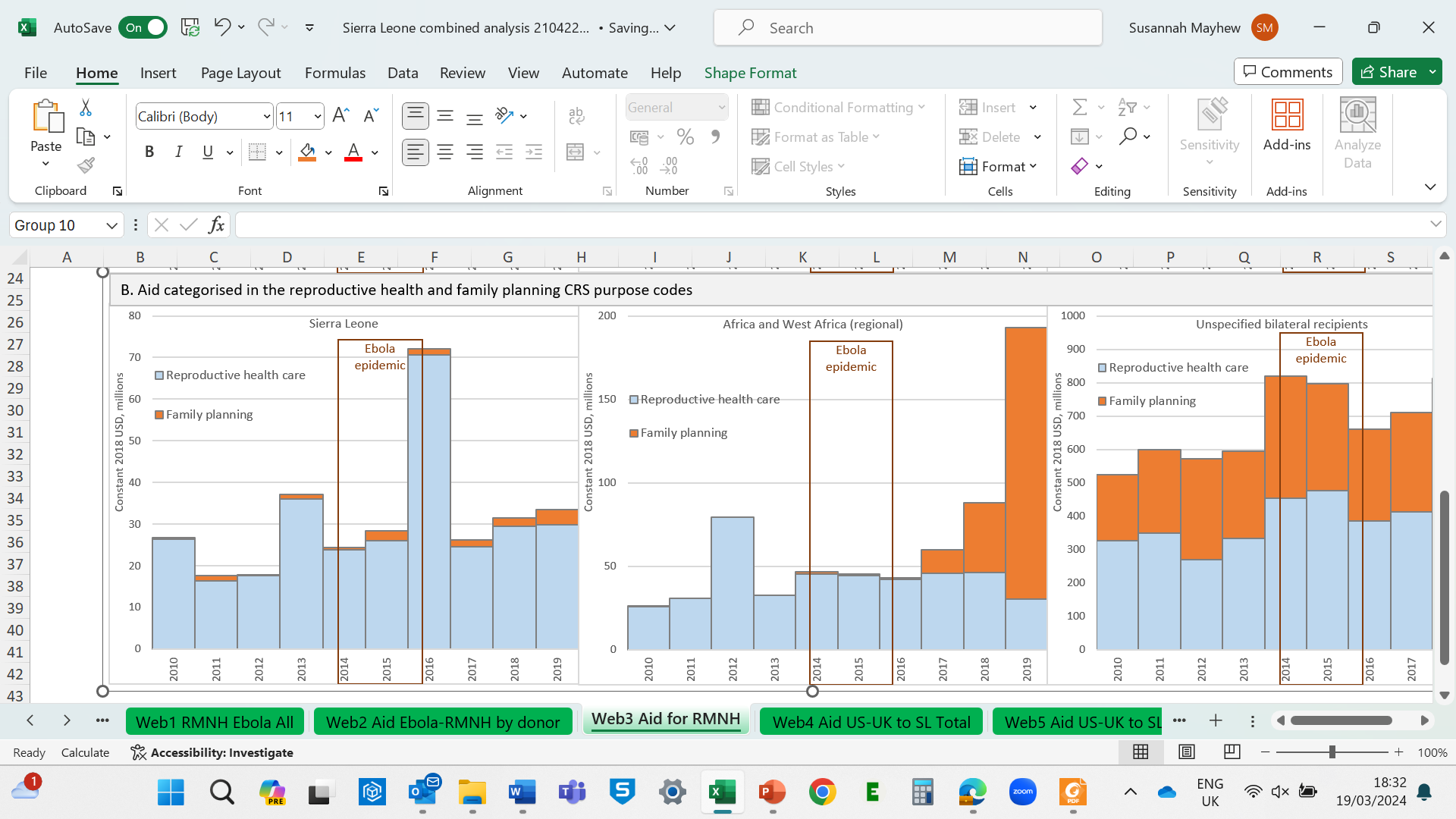

Supplement: Supplementary file 1 — Additional file 1: Annex 1. List of records excluded from Ebola categorization. Annex 2. Detailed breakdown of all sector data: aid to Sierra Leone, Africa and West Africa region. Fig. S1. Total aid for all sectors by sector. Fig. S2. Total aid to Sierra Leone, Africa and West Africa, 2010-19: Ebola, RMNH, and other purposes. Fig. S3. Aid for Ebola by sector. Fig. S4. Aid for Ebola and RMNH to Sierra Leone, & Africa/West Africa, by donor (top 5), 2010-2019. Fig. S5. Aid for RMNH to Sierra Leone and Africa/West Africa, 2010-2019, disaggregated by components. [file 13031_2024_589_MOESM1_ESM.docx]
